# Bibliotherapy: Reading OVID During COVID

**DOI:** 10.3389/fpsyt.2020.567539

**Published:** 2020-12-07

**Authors:** Emmanuel Stip, Linda Östlundh, Karim Abdel Aziz

**Affiliations:** Department of Psychiatry, College of Medicine and Health Sciences, United Arab Emirates University, Al Ain, United Arab Emirates

**Keywords:** pandemic (COVID-19), psychotherapy, bibliotherapy, stress, depression, medical literature

## Abstract

An epidemic of an infectious disease such as COVID-19 is often a source of emotional distress, even among those who have not been directly exposed to the disease. The period following the acute phase of the coronavirus epidemic and the mitigation measures will likely be hardest for medical professionals in terms of psychological impact. Bibliotherapy is a systematic intervention regarding the use of carefully selected reading materials in order to help persons to cope with stress and personal problems. This therapy can be used easily during the pandemic. The review of evidence shows that this kind of intervention can be helpful in educational and clinical contexts. During the crisis, it can be an alternative to video and film entertainment and a transition from serious medical journal clubs to a softer medical humanities experience. In this article, we summarized the historical background of bibliotherapy. We also proposed a reading list from different times, and cultures relating to pandemic, quarantine, symptoms, confinement, and social impacts (e.g., Camus, Moravia, London, Le Clezio etc.). Bibliotherapy can be a way for doctors and healthcare workers fighting on the frontline of the pandemic to find psychological support and for debriefing. Bibliotherapy can help individuals that need support for emotional distress during the pandemic to verbalize their feelings and emotions and identify new ways of addressing problems.

## Introduction

Some of us are in exile, far from our families, trapped and confined, sometimes in quarantine or starting a risky de-confinement (1–3). Remote work is possible. Television is accessible with series and continuous information. And sometimes when we choose television and are tired of counting deaths by country or county, we watch action films. And there, we no longer count the deaths and the machine-gun shots wielded by heroes rescued from so many shots and explosions. So we lean toward reading as a way of healing and not counting anymore. Sometimes, in our exile, we are not lucky to have taken a library in our suitcases with the chosen books and in a familiar language. Fortunately, there are electronic libraries and e-books. And there, we start reading and dreaming. And we are rediscovering Ovid, our alternative to Covid. Ovid, in Latin Publius Ovidius Naso, is a Latin poet who lived during the period which saw the birth of the Roman Empire. He too had to exile or confine himself to an island. The reasons for his exile remain mysterious: the subversive remarks made in “The Art of Loving” is the main reason cited by critics. One of us (ES) was able to find and read it and it was a therapy, a bibliotherapy.

This article emphasizes the importance of bibliotherapy during the COVID-19 crisis. In this article, we propose to reposition bibliotherapy as a means of treating psychological distress, boredom, isolation, and limitations during the pandemic. Bibliotherapy can be a way for doctors and healthcare workers fighting on the frontline of the pandemic to find psychological support and for debriefing.

Confinement weighs on us and deconfinement stresses us (4, 5). Every book we love is a treasure. A window on a world, fantastic, dream-like, whacky, childish, foreign, from another era, which one then has either jealously stored in their library or carelessly buried in the back of a closet. But some volumes are even more than that. They are talismans, protective cocoons, in which we delve back into delightful times. We re-read them to soften our spleen, soothe our panic, or escape from an everyday life that has become too heavy.

## Bibliotherapy

Bibliotherapy is a form of clinical or self-developing therapy, often used in connection to psychotherapy, that includes reading as a part of a treatment (6, 7). More specifically, therapeutic reading is a source of appeasement for mental health disorders (namely, anxiety disorders, mood disorders, depressive episodes, phobias, sleep disorders, etc.) or for strengthening psychological well-being (8–12).

In the midst of the First World War, doctors and booksellers at the Alabama Military Hospital in the United States used books to relieve posttraumatic stress disorder for soldiers returning from combat (13). Then, the 1950s marked the proliferation of research on bibliotherapy in a wide variety of fields (nursing, social work, education, etc.), mainly in North America, to which booksellers largely contributed. In 1961, the definition appeared in Webster International: “bibliotherapy is the use of a set of selected readings as therapeutic tools in medicine and psychiatry; and a way to solve personal problems through directed reading.” In the 1970s, experiences multiplied and really rubbed shoulders with the mental health sector in North America and Europe (13). Care through reading applies to the elderly as well as to individuals suffering from eating disorders or childhood disorders. The experiences are numerous but nevertheless *ad hoc* and emanate from localized initiatives, often within hospital libraries. In this context, the trend cannot flourish on a larger scale because no reproducibility has been observed or implemented from one project to another (13).

Three categories of books used in bibliotherapy are identified (14): the classical repertoire (novel, poetry, biography, fiction) which, often by a process of identification, brings better well-being to the patient, and works whose theme is psychology—their approaches are varied; they can describe a current disorder as well as provide information on a specific disorder. These specifically aim to assist readers. There is a fine line between this category and the last, coined as “self-help books,” to be linked to so-called cognitive development publications of personal inspiration, offering a precise methodology to relieve a malaise.

## History

It was religious texts that came to be the most used literature for early bibliotherapy up until the mid-nineteenth century (15). The first known organized form of bibliotherapy in clinical settings can be dated back to thirteenth century Egypt where the hospital staff and religious leaders at the Al Mansour Hospital in Cairo read the Quran to their patients in addition to medical treatment (16, 17).

The therapeutic benefit of reading was first identified by the ancient Romans and Greeks and can be found in theories by well-known scholars and thinkers throughout history (17, 18). In “Poetics,” the Greek philosopher Aristotle (384–322 BC) presented the concept of using literature and drama for healing and purification (catharsis) of negative emotions. Later, the philosopher Friedrich Nietzsche (1844–1900) and the neurologist Sigmund Freud (1856–1939) referred to Aristotle's idea of catharsis when describing how literature can have a therapeutic effect on negative emotions (19). Similar connections between literature and therapy can also be found in the architecture of some of the earliest known libraries. The inscription “The House of Healing for the Soul” was, according to the Greek historian Diodorus Siculus (9–30 BC), written over the entrance to Ramses II library in Thebes, Egypt, around 300 BC (16, 20–23), and the inscription “apothecary of the soul” can be seen in the medieval library of the Abby of St. Gall in Switzerland (20). Inspired by Freud's work, fairytales, symbols, and myths became a central theme within the Jungian psychoanalytic context in the mid and late twentieth century (22). Key publications such as Carl Gustav Jung's “Man and his Symbols” (23), Marie Louise von Franz's “Interpretation of Fairy Tales” (24), and the psychotherapeutic and gender political books “Iron John: A Book About Men” by Bly (25) and “Women Who Run with the Wolves: Contacting the Power of the Wild Woman” by Estés (26) are a few examples of how fairytales and myths can be used for self-development.

In addition, according to some authors, Bibliotherapy can be called developmental bibliotherapy (17, 27), or affective bibliotherapy (28) to differentiate it from cognitive behavioral bibliotherapy (CBT). CBT mainly uses self-help books (9).

## Current Research and Practical Framework

In modern clinical or developmental bibliotherapeutic settings, mental health professionals may prescribe selected fiction or non-fiction materials such as novels, short stories, biographies, dramas, tales, fables, and poetry as a part of a patient's treatment (17). A study by Bruneau and Pehrsson emphasizes the importance of choosing personalized reading materials and encourages bibliotherapists to involve their clients in the selection process as an opportunity to foster self-insight and motivation to read (28). The growing and increasingly popular self-help books are an additional form of bibliotherapy that can be used in conjunction with cognitive behavioral therapy (17). Depression, anxiety (29), posttraumatic stress disorder (30), panic attacks (31, 32), insomnia (33) and stress (34), and strokes and their psychological consequences (35) are some examples of psychiatric and psychological conditions where self-help books have been proven to be helpful.

A study published in PLOS One showed convincing results for the effectiveness of this form of care. The Scottish research team brought together more than 200 patients diagnosed with depression; half were put on antidepressants while the other followed a therapy program through reading the book “Overcoming Depression” and having related discussions with psychologists. At the end of 4 months, 42.6% of patient-readers saw their degree of depression reduce significantly compared to 24.5% of patients on medication. After 1 year, they were more able to manage depression than the other group (36). However, this study was conducted using a guided self-help CBT treatment, whereas our attempt to currently review literature on bibliotherapy is more about another category (mainly the “classical” repertoire such as novels, poetry, and biographies) and not works whose theme is psychology. One of us (ES) used a book, “Address Unknown,” a novella written by K. Kressmann Taylor, to assess the theory of mind and to propose a cognitive remediation for patients with schizophrenia. Simply reading a 20-page novella became a cognitive task, with a good ecological component (37).

Recent studies from Sweden (2020) and Poland (2017) indicate that bibliotherapy can be an efficient complement to therapy and traditional medical interventions (38, 39). However, more clinical studies are needed to support physicians and psychologists with an evidence-based framework for clinical bibliotherapy (10, 29, 34, 39).

Bibliotherapy was originally developed to treat depression. It has also been used among caregivers in recent years (40).

## Caregivers and Health Teams

The effectiveness of bibliotherapy for caregivers has been achieved through a series of different studies. The results of these studies suggested that bibliotherapy was effective in improving the care experiences of caregivers of people with psychosis (40), as well as the resilience of caregivers who care for people with depression (41).

Several studies, including a meta-analysis, using bibliotherapy to improve the mental well-being of caregivers with neurocognitive disorders have also been considered and suggest a favorable effect on their well-being (8, 42).

With new technology and easy access to literature through online libraries and bookshops, bibliotherapy has become an efficient and inexpensive alternative to traditional face-to-face therapy (29). Self-paced reading with follow-up sessions over the phone, videoconferencing, or being in virtual reality settings enables individuals with economic, geographical, physical, or mental barriers to benefit from bibliotherapy (17). The new online or telemedicine options for bibliotherapy have the potential to work very well in pandemic and self-isolation settings.

With the pandemic, overexposure to the stressful news magnifies the feeling of threat, becomes a waste of time, and can even paralyze the individual, preventing them from protective behaviors and facing life demands. Mental health professionals may treat patients with increased emotional distress caused by the effects of the pandemics on them, on their families, or on their community. Several recent studies highlight the psychological impact of COVID-19 and the need for guidelines and increased psychological interventions (43–45). Depression, anxiety, and insomnia are some examples of psychological distress found among the general population as a result of self-isolation, social distancing, and safety concerns (43–45). The period following the acute phase of the coronavirus epidemic is the hardest for medical professionals in terms of psychological impact (46, 47).

Despite a call for more evidence-based research about the wider effectiveness of bibliotherapy (11, 18, 30, 39, 48, 49), the reported benefits and zero harm makes bibliotherapy an effective form of therapy for individuals with mental and personal development issues (30, 49). Bibliotherapy can be useful for health professionals and physicians. Prescribing a “transitional book” means that the content is part “microbes” and part “magical realism.”

## Proposal: From Journal Club to Bibliotherapy

The information explosion in the pandemic era poses a challenge on how to extract useful resources from a multitude of publications on a daily basis. Doctors and healthcare professionals are bombarded with data. The publication rate is exponential. There are already academic structures for sorting and synthesizing literature. A journal club is an effective approach to tackle these problems; therefore, it has already become an integral part of university education in almost all medical specialties. A journal club is a form of meeting regularly organized between health professionals to discuss related recently published literature. The first organized newspaper club was awarded to Sir William Osler in Montreal, Canada, in 1875, although Sir James Paget described a kind of club among some students at St Bartholomew Hospital in London to read newspapers together between 1835 and 1854 (50). Several decades later, Osler started the first journal club in the United States at Johns Hopkins Hospital in 1889. Over the next 100 years, journal clubs flourished in various medical disciplines in many countries (50).

One of the great challenges of medical education these days is the efficient selection and refinement of relevant literature from a plethora of available information. Journal club formats have evolved over time. However, with this pandemic, physicians, students, and health professional are overloaded and need to find a moment of relaxation where the knowledge can be exposed differently. In addition, exposure to so much data is very heavy. This can be a factor in increasing pressure or stress on mental health. We must arrange different times or ways of doing things to also allow an escape while reflecting on the area around us. The literature-based reading exercise can be an alternative to the austere conference of scientific articles.

The book can be a pretext for sharing experience and novels, comics, and other literary fiction invite you to escape. In these pandemic times, it may be necessary to change or also alleviate the minds of professionals and the public by offering transitional books, i.e., that concern the subject but allow a distance and a new hedonism in the company of a book.

## Potential Reading List

The following are some examples of bibliotherapy that one of us (ES) prescribed to their colleagues:

**The Plague** by Albert Camus has been acclaimed in Italy since the start of the pandemic. In France, sales of this novel have exploded since its appearance. In terms of containment, it ranks among the top 20 best sellers of digital books. “What you learn in the midst of the plagues is that there is more to admire in men than to despise” writes Camus. This novel, which takes place in the 1940s in Oran, Algeria, begins when a strange disease kills very large numbers of rats, then humans. Camus used an allegory to speak of evil, of everything that oppresses us that we have to fight against. This use of allegory means that a 2020 reader can find himself completely and project the coronavirus onto it. The novel lists the multiple reactions of a community to the epidemic: the authorities which are slow to react, the underestimation of the danger, the containment measures, the solidarity which is being put in place but also the profiteers who are getting richer thanks to the black market, etc. Dr. Rieux, the central figure in the novel, reflects the committed, courageous, and generous medical personnel on the front line in the fight against the epidemic. The hero of the story is a modest office worker called Grand. This character would like to write a novel and spends his time repeating the first sentence. At the start of the novel, a journalist called Rambert has only one idea in mind: to join his fiancée who is outside the city. Initially he seeks to flee as someone who today would rather follow his personal desire than obey the common good and accept this confinement which is difficult. Little by little, he will enter the solidarity movement. But as the days go by, he begins to fear that this misfortune will have no end and, at the same time, the cessation of the epidemic becomes the object of all hopes. The end of “The Plague” encourages us not to forget too quickly what we have experienced. Camus reminds us that we must not “forget what we experienced, the unhappiness that happened to us, and everything that has taken place in our capacity to be united in times of trial, to get out of our selfishness.”

**Love in the Time of Cholera**. At the end of the nineteenth century, in a small Caribbean town, Florentino, a poor young telegraph operator and a lovely schoolgirl, swore to marry and live in eternal love. For 3 years they live together, but Fermina marries Juvenal Urbino, a young and brilliant doctor. So Florentino, the betrayed lover, turns into an unrepentant seducer and strives to make a name and a fortune to deserve the one he will never cease to love, in secret, lasting for 50 years, until the day when love will triumph. The analogy proposed by the Colombian writer Gabriel García Márquez between the epidemic and the amorous passion which shakes beings is particularly deep and conducive to reflection. Cholera is not the central subject of García Márquez's book, published in 1985, 3 years after being awarded the Nobel Prize for literature. Evil, as with Camus, is invisible. It strikes this one or that one, according to its pleasure and according to the rules of love and chance. But with Camus, the epidemic was a metaphor for war. With García Márquez, it is closer to a loving “passion” that sneaks into the body, nourishes the same symptoms, grows and shakes the body.

**Geopolitique du Moustique** (Mosquito Geopolitics): Following in the footsteps of mosquitoes to write this book, Erik Orsenna has traveled to some of the countries where the diseases transmitted by mosquitoes are rife. In addition to his travels, there are regular visits to the Institut Pasteur in Paris. You only learn about yellow fever there. About 27,500 people died on the two shipyards, French and American, of the Panama Canal between 1882 and 1914, in particular from yellow fever without anyone really knowing what this epidemic was. It was only at the beginning of the twentieth century that we were really able to discern the causes and that the Americans, successors of the French, resolved this problem, both major and unforeseen, which did not exist in Suez. Yellow fever brought its share of corpses every day, and gave the survivors a feeling of precariousness.

**Death in Venice**. At the beginning of the twentieth century, a famous writer wrote an astonishingly topical text in these days of confinement. In his short story Death in Venice, Thomas Mann has indeed described the process which leads tourists to be caught in the cracks of which they can hardly root out. Beyond his masterful literary text and his intrigue which sees a writer, Gustav Aschenbach capturing a mad passion for a teenager, he works as a very discerning observer by showing the sneaky diffusion in Venice of what he called “Asian cholera.” Mann's text helps to see how the epidemic has spread in Europe and particularly in Venice and how it is affecting the city and its people, especially tourists. We complete the list which is not exhaustive in the Table 1.

**Table 1:** A subjective collection of potential books covering topics relevant during times of the COVID-19 pandemic.

**Titles**	**Authors**	**Themes**	**Commentaries**
The Plague (*La Peste*)	Albert Camus	Epidemic Plague	The characters of *La Peste* are lucky to be able to circulate in Oran, in Algeria during the ongoing plague Camus reminds us that it's important to have moments when you recharge your batteries and regain your strength at the heart of the plague
Love in the Time of Cholera	Gabriel García Márquez	Epidemic Cholera	Cholera epidemic forms the backdrop to a doomed love story, with an analogy being made between the epidemic and amorous passion. The cholera randomly strikes at its pleasure like the rules of love and chance, sneaking into the body, and nourishing the same symptoms
Death in Venice	Thomas Man	Epidemic	The author describes a process which is divided into several stages: 1. Asian origin of the epidemic −> 2. Arrival of the epidemic in Europe −> 3. Identification of the “zero patient” −> 4. Transmission of the epidemic −> 5. Events −> 6. Measures taken by the authorities −> 7. Reactions from the public −> 8. Implications (departure or confinement)
The Perfume	Patrick Süskin	Olfaction	Loss of smell can be the first symptom of COVID. Süskind has put an anti-hero at the center of his action. Ironically, he throws Jean-Baptiste Grenouille, a Parisian orphan, into the world of smells. He can perceive odors from afar and dissect them. He's a stranger with a unique talent and a murderous idea. Grenouille wants to create the largest of all perfumes to finally be part of the world
Geopolitics of the Mosquito (*Géopolitique du Moustique*)	Erik Orsena	Infections Yellow Fever	The precariousness experienced by survivors of Yellow Fever epidemics in the early Twentieth Century is explored and how it incited self-destructive behaviors such as gambling, drugs and prostitution
Little Women or Meg, Jo, Beth and Amy	Louisa May Alcott	Epidemic Scarlet fever	Scarlet fever is caused by group A streptococcus, transmitted through the air and most often from an affected child (a sore throat, inflammation of the tonsils, and small scarlet red spots). Treatment is based on antibiotics One child character died from complications which was contracted after she visits the sick children of an impoverished neighbors
The Lady of the Camellias	Alexandre Dumas	Epidemic Tuberculosis	Climbing the ranks of prostitution in record time, the heroine, passed in a few months from misery to fortune. Pulmonary tuberculosis had a venereal connotation in the nineteenth century The novel was then adapted for the stage and performed at the Vaudeville theater before inspiring Giuseppe Verdi to play the character of Violetta in La Traviata
The Masque of the Red Death	Edgar Poe	Epidemic	Poe highlights the principle of the equality of all humans in the face of death. Prince Prospero, the main character favored the knights and ladies of his court who resolved to barricade themselves against the sudden impulses of external despair. This protectionism operated to the detriment of the people kept outside the walls Poe draws a distinction between masque and mask Masque as expressing a dance, a masquerade, a farandole composed of masked characters
The Scarlet Plague	Jack London	Epidemic	Jack London speaks of the carefree people spared by the disease as they watch it spread to other regions, never imagining that it will 1 day reach their own
The Stand	Stephen King	Epidemic	Stephen King dedicates several chapters of his long novel to the transmission of the virus, by characters who do not know that they are already sick
Station Eleven	Emily St. John Mandel	Epidemic	The author describes the collapse of our societies in the face of a lightning flu
The Epidemic (*L'epidemia*)	Alberto Moravia	Epidemic	The book is a satirical text on fascism
The Horseman on the Roof	Jean Giono	Epidemic Cholera	The hero, an Italian hussar from Piedmont, is on the run after winning a deadly duel. His tribulations lead him to Manosque, in Provence, where a cholera epidemic is raging. Pursued by the authorities, who believe him guilty of poisoning the city's fountains, he wanders over the roofs of abandoned houses. Armed with inexplicable immunity and noble devotion, he put himself at the service of a few convicts in the hope of saving them
The Quarantine (*La Quarantaine*)	Le Clézio	Quarantine Smallpox	On a ship, passengers develop symptoms of smallpox. All are forced to land on Flat Island, a volcanic haven in the Indian Ocean, where they will have to remain in quarantine for an indefinite period. Le Clézio reports in *La Quarantaine* the experience of forced isolation, on an island where colonization separates Europeans from Indian immigrants hired to work in the colonies
In a Perfect World	Laura Kasischke	Epidemic Influenza	The epidemic story is a tool to paint a vitriolic portrait of the American middle class
Nemesis	Philip Roth	Epidemic Poliomyelitis	Philip Roth peels at the emotions aroused by the fury of an epidemic
Peloponnesian War	Thucydide	Epidemic Plague	The plague of Athens devastates Greece and describes the anarchy which is spreading in the city with the disease
The Last Man	Mary Shelley	Plague Pandemic	The author asks her readers to imagine a world in which only humans are becoming extinct. Attacked by a new, unstoppable plague, the human population collapses within a few years. Other species flourish. A rapidly decreasing band of survivors watches as the world begins to return to a state of conspicuous natural beauty, a global garden of Eden
A world without female (*Il mondo senza donne, Italian*)	Virgilio Martini	Unspecified virus	Conceived in the secrecy of a laboratory, at the beginning of the twenty first century, by a club of American homosexuals who have sworn the disappearance of women, the virus attacks only the weak sex, between puberty and menopause, and mows down its first victims in Haiti. Over time, the demographics are collapsing
All Fools' Day	Edmund Cooper	Radiations Mental illness	Only the eccentrics, the obsessed, creative artist, fanatics of various kinds and psychopaths seem to be immune and survive this mysterious radiation
The Ninth Day (*Le neuvieme jour, French*)	Hervé Bazin	Influenza “super-flu”	This describes a society which flirts between the benefits of technical and technological progress and the dangers, which allows a reflection on science and the new role of men
The Eyes of Darkness	Dean Koontz	Unspecified virus	Dean Koontz novel describes a killer virus developed in a Chinese bioweapon lab called “Wuhan-400.” First published in 1981 under a pseudonym, the virus, originally produced in Russian, became Chinese in a 1996, post-cold war revision of the book
Plague	Graham Masterton	Epidemic	Department of Public Health support the thesis of the ephemeral phenomenon, but the situation is deteriorating. It is a very contagious fatal disease, the virulence of which is increased 10-fold by an unknown mutation Little by little, the city sinks into chaos. The American authorities use radical methods to contain the epidemic
Pandemic 1918	Catharine Arnold	Pandemic Influenza	Rapid spread of the disease as infected populations were shifted in the wake of the war, the vicious nature of the “second wave” of the disease Spanish flu was that it often struck the healthiest rather than the elderly, young or weak

In Japan, Amabie, a legendary creature whose image is supposed to protect against epidemics, has emerged from oblivion since the Covid-19 crisis. In early March 2020, a tweet about this from the Kyoto University Library went viral and triggered an “Amabie-challenge” where many artists began to draw Amabie and publish their works on social networks. This helped foster creativity in a large network, an essential element of resilience.

## Conclusion

In this article, we presented the historical background of bibliotherapy and then suggested some books by authors, which can be used independent of culture. Even if reading does not replace a session with the psychologist or therapist, scientific studies have proven the many benefits of reading: it reduces stress, improves the quality of our sleep, and stimulates emotional intelligence. Even if it is rather a solitary activity, reading can really facilitate human relationships by making you more empathetic, provided you feel overwhelmed and transported by the story of the novel you are reading. Reading improves memory and involves memorizing to keep the plot in mind, as well as the names of the characters and their relationships. During this pandemic and in anticipation of several new waves, it is necessary to create strategies and propose concrete therapies favoring groups constrained by social distancing but able to exchange content from a book with a therapist. This review describes bibliotherapy as a means of coping with the extraordinary situation of the COVID-19 pandemic and the associated challenges for mental health. We highlighted that bibliotherapy can be helpful to support people (mainly frontline workers) who are under emotional stress or suffering from mental illness. We provided a historical overview of bibliotherapy and also examples of potential books covering topics relevant to the times of the COVID-19 pandemic.

## Limitations

The limitation of this article is that it reflects subjective views rather than statistically sound evaluated findings. The literature for the evidence for the efficacy of bibliotherapy is still heterogeneous but is more homogenous in the field of education, school, etc. There are some studies in depression but very few well-controlled studies that often include the bibliotherapy as a part of CBT. A meta-analysis is still possible but difficult at this stage (51–54). For instance, the effects on reducing depression should be still viewed with caution due to high heterogeneity. The effects on other mental well-being outcomes are inconclusive due to limited number of studies, and this underscores the need for further research. The selection of these literary works is also limited to a certain western culture. It would be necessary to extend it to other cultures, Asian, African, Amerindian etc.

The COVID-19 pandemic is still raging, and it is very likely that, for a relatively long time, we will have to live with it. It is the evolution of the global health situation that will dictate how we operate, its progress, and its possible setbacks. Caution is required in our collective decisions as in our individual behaviors, and the responsibility for each and every one is engaged here. We have listened to science from the start, and that is what we will continue to do in the weeks and months to come. We must also create moments of escape because our physical space to travel is no longer the same. Imaginary trips can be shared, through the literature. Bibliotherapy is a way to structure these trips in order to improve mental health resilience.

Even if containment or social limit measures are in place, technology with ZOOM, MS teams, Blackboard, etc., allows the realization of meetings based on the effects of reading on each of us.

## Author Contributions

ES wrote the first version. LÖ and KA reviewed it and added complementary section. All authors contributed to the article and approved the submitted version.

## Conflict of Interest

The authors declare that the research was conducted in the absence of any commercial or financial relationships that could be construed as a potential conflict of interest.
